# Bioavailability factor for improved drinking water contaminant exposure risk assessment accuracy

**DOI:** 10.1016/j.fmre.2024.10.010

**Published:** 2024-11-01

**Authors:** Yayun Zhang, Shengkun Dong, Di Zhang, Michael J. Plewa, Wenhai Chu

**Affiliations:** a State Key Laboratory of Pollution Control and Resources Reuse, College of Environmental Science and Engineering, Tongji University, Shanghai 200092, China; b Shanghai Institute of Pollution Control and Ecological Security, Shanghai 200092, China; c Guangdong Engineering Technology Research Center of Water Security Regulation and Control for Southern China, School of Civil Engineering, Sun Yat-sen University, Guangzhou 510275, China; d Department of Crop Sciences, University of Illinois at Urbana-Champaign, Urbana, IL, 61801, USA; e Safe Global Water Institute, University of Illinois at Urbana-Champaign, Urbana, IL, 61801, USA

**Keywords:** Drinking water, Chemicals, Bioavailability factor, Risk assessment, Biological retention ratio, Emerging contaminant

## Abstract

Despite being low in concentration, the presence of broad-spectrum chemical constituents in drinking water is a fact of modern life. The current drinking water chemical risk assessment practice predominantly relies on drinking exposure and chemical toxicity data, providing a valuable tool for regulation. However, only a fraction of chemicals is retained for a considerable duration within the human body, as the remainder are excreted or metabolized. This limits the efficacy of risk assessment when exclusively relying on water contaminants ingestion dose and toxicity potencies. We propose large scale incorporation of a bioavailability factor, *F*, into the calculation of chemical-mediated human health risk assessment for drinking water. Specifically, *F* values account for the ratio of one compound in human circulation and excretion and are multivariable specific, such as exposure route specific, and gender specific. Utilizing disinfection by-products (DBPs), an important group of chemicals commonly found in drinking water as an example, we provided detailed explanations of *F* values. Through incorporation of the *F* values, the accuracy of drinking water human health risk assessment may be improved as the biological retention ratio is accommodated, providing leniency on chemicals that less interact with the human body during internal retention. This approach has clear implications for the subsequent control measures and national drinking water standards updates for emerging pollutants.

## Graphical abstract

**Figure:**
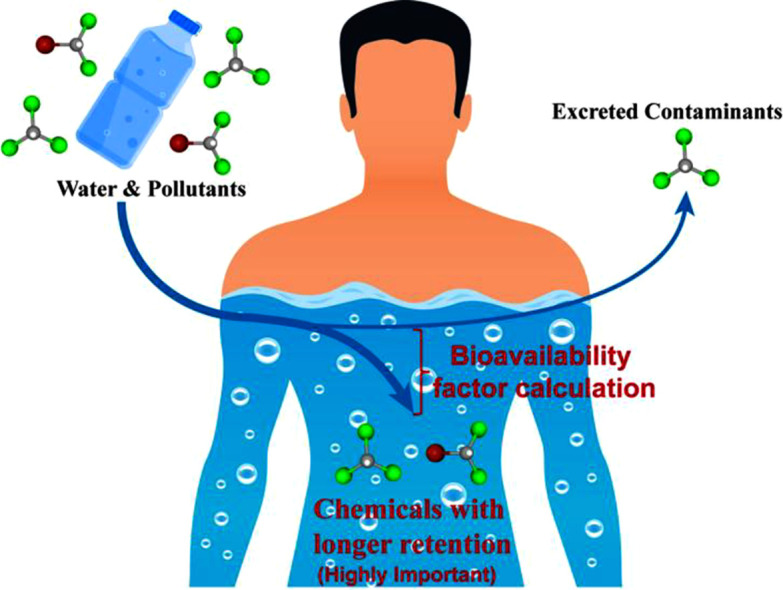

Broad-spectrum chemical constituents, such as pesticides, antibiotics, and disinfection byproducts (DBPs), are widely detected in drinking water globally, posing potential threats to terminal users. For instance, low concentration exposures to neonicotinoid insecticides could induce genotoxicity, cytotoxicity, impaired immune function, reduced growth, and reproductive failures in animal studies [1]. As another example, exposures to certain DBPs that are unintentionally generated in water treatment, have been shown by epidemiological studies to demonstrate positive correlations with increased risks of bladder cancer, birth defects, and miscarriage [[2], [3], [4]].

There are several routes of human exposure to chemicals in drinking water, including ingestion through non-potable applications (e.g. dermal, and inhalation), and ingestion during swimming [5]. After drinking exposure events, biological processes play pivotal roles to rid of these chemicals, such as via metabolism, sweating, and urination. As an example, DBPs are frequently detected in biological samples (e.g. urine, breath), including trihalomethanes (THMs) (detection frequencies, (DF = 88%∼100%) and haloacetic acids (HAAs) (DF = 100%) [6,7]. Therefore, the actual retained fraction of drinking water chemicals within the body available to induce adverse biological impacts may be lower than that ingested or directly exposed. This suggests lower health risks from lower concentrations of pollutants than extrapolated from direct exposure. This concept presents a new challenge to quantify the human exposure risks to chemicals in drinking water.

To address the above problem, we propose applying the concept of ‘bioavailability factor (*F*)’ to the calculation of the drinking water chemical-mediated human health risks. The *F* factor is expressed below:

(1) $$F=\frac{D-E}{D}$$

where *F* is the bioavailability factor of the corresponding chemical; D is the original chemical exposure dose in drinking water upon oral intake; E is the excretion of experimental targets from urine and sweat.

Similar concepts have been applied to calculate the absorption rate of multiple compounds in the circulatory system (1), such as drugs (e.g. berberine ) [8], nutraceuticals (e.g. beta-carotene) [9], and pollutants (e.g. pesticides) [10]. As a fraction of chemicals are retained in the body, this factor is suitable for assessing the retained concentration of chemicals in humans, factoring in the excretion process. Bioavailability factor *F* can be seen as the compound retention ratio between the in vivo vs. in vitro experiments, and may be influenced by a multitude of factors, such as structure types (e.g. nitrogen-containing DBPs vs. carbonaceous DBPs) and exposure routes (e.g. inhalation vs. dermal absorption) [11]. Physiological differences in humans, such as age, gender, or underlying diseases, could also affect *F* values [12]. This approach requires comprehensive investigations into the *F* values for individual contaminants.

Chemicals with higher *F* factors are enriched in target organs, such as 2,4,6-tribromophenol that accumulates in the muscle tissue of the European conger eel, and the sexual gland of the purple sea urchin [13]. Chemicals with lower *F* factors are excreted from the body more easily, leading to lower average circulatory concentration than ingested with shorter contact time with susceptible target organs. It should be noted that some chemicals that are easily excreted may also induce toxicity responses by inhibiting critical cellular enzymes before being excreted in their original form, such as bromoacetic acid and iodoacetic acid [14,15]. Such chemicals need to be identified and possibly assigned higher F values than that typically calculated by Eq. 1, the extent of which depends on experimental observation that reflects the deviation of the chemicals' adverse effect from the average situations. The difference between the *F* value-corrected concentrations vs. the ingested total dose may represent the drinking water chemical fraction that contributes only limited adverse health effects. A low retention fraction should be regarded as having less importance to the induction of adverse health risk. The focus for health risk evaluation should be directed to chemicals that are more bioavailable and more toxic. This would modify each chemical's concentration-weighted toxicity for the widely adopted component in the model for the chemical risk assessment of drinking water. It would be useful to adopt the previous drinking water chemical-associated health risk assessments and attempt to improve the accuracy of the predicted risks by incorporating the following expressions such as the TI (toxicity index, 2). Adjustment of F values refines the TI of certain contaminants, thereby providing a more accurate assessment of their risk in drinking water. For example, risk assessment on example contaminants was employed and re-calculated by adopting parameters including the bioavailability, water concentration, and LC_50_ (Table 1). As is shown in Table 1, the risk of TCA exposure was underestimated (0.70 × 10^–3^ to 0.81 × 10^–3^), while DCA (0.58 × 10^–3^ to 0.47 × 10^–3^) and DBA (0.82 × 10^–3^ to 0.25 × 10^–3^) were both overestimated after bioavailability correction.

(2) TI=Tc×Cx×F

where TI is the toxicity index of contaminants; Tc is the reciprocal of the chemical's LC_50_ values (or similar values for various endpoints); Cx is the chemical concentration in water; *F* is the bioavailability factor of each chemical in fraction. The TI calculation of compounds was primarily focused on their main exposure routes to humans rather than other minimal F values contribution.

**Table 1:** **Calibration of risk assessment of some example contaminants by *F* values**.

Contaminants	Trichloroacetic acid	Dichloroacetic acid	Dibromoacetic acid
Abbreviation	TCA	DCA	DBA
Bioavailability (%)^a^	116	81	30
Concentration (µg/L)^b^	1	2	2.4
LC_50_ (µM)^c^	1430	1720	1220
Toxicity data (10^-3^)	0.70	0.58	0.82
Adjusted toxicity data (10^-3^)	0.81	0.47	0.25
Risk assessment	Underestimation	Overestimation	Overestimation

a Originated from (I. R. Schultz, et al., 1999).

b Originated from (J. M. Allen, et al., 2022).

c Originated from (Y.-T. Zuo, et al., 2017).

In summary, the concentration of drinking water chemicals in the human circulatory system is always lower than that taken in via different exposure routes. The internal concentration difference is dependent upon biological processes such as metabolism and excretion. Chemicals with higher *F* values exert longer exposure durations in the circulatory system and accumulate in targeting organs than those with lower *F* values. Incorporation of *F* values into the drinking water chemical risk assessment more accurately prioritizes pollutants that are of top concerns to be treated in drinking water treatment. Admittedly, a generic *F* value for any compound is indeed challenging at this stage because the fluctuation of *F* values across different compounds, exposure routes, and physiological conditions. In light of this, we propose a phased approach for a more universally applicable *F* factor following as 1) several statistical models were to predict a reasonable average *F* value of priority chemicals (e.g. random forest models, graph nerual network, convolutional neural networks). This calculation comprehensively incorporated various aspects, including chemical structure, exposure pathways, and physiological elements; 2) high *F* values with selected contaminants will be screened and revised accurately by in vivo models (animal models, pharmacokinetics model). This *F* evaluation needs to be comprehensively considered with contaminants and relevant tranformaiton products (e.g. progressive untarget screening); 3) the refined bioavailability model will predict *F* values of non-priority contaminants, and then, targets with high *F* values were also point out; 4) among that, the toxicity risk evaluations of high *F* values all contaminants will be re-calculated accurately in drinking water. Our approach has clear implications for the subsequent control measures and national drinking water standards updates for emerging pollutants. Enhanced collaborations among environmental scientists, toxicologists, chemists, engineers, and regulators are needed to calculate and integrate *F* values of different drinking water chemicals into existing drinking water chemical risk assessment models.

## Declaration of competing interest

The authors declare that they have no conflicts of interest in this work.
